# AMGDTI: drug–target interaction prediction based on adaptive meta-graph learning in heterogeneous network

**DOI:** 10.1093/bib/bbad474

**Published:** 2023-12-25

**Authors:** Yansen Su, Zhiyang Hu, Fei Wang, Yannan Bin, Chunhou Zheng, Haitao Li, Haowen Chen, Xiangxiang Zeng

**Affiliations:** Information Materials and Intelligent Sensing Laboratory of Anhui Province, Anhui University, Hefei, 230601, China; Information Materials and Intelligent Sensing Laboratory of Anhui Province, Anhui University, Hefei, 230601, China; Information Materials and Intelligent Sensing Laboratory of Anhui Province, Anhui University, Hefei, 230601, China; Information Materials and Intelligent Sensing Laboratory of Anhui Province, Anhui University, Hefei, 230601, China; Information Materials and Intelligent Sensing Laboratory of Anhui Province, Anhui University, Hefei, 230601, China; Information Materials and Intelligent Sensing Laboratory of Anhui Province, Anhui University, Hefei, 230601, China; College of Computer Science and Electronic Engineering, Hunan University, Hunan, 410082, China; College of Computer Science and Electronic Engineering, Hunan University, Hunan, 410082, China

**Keywords:** drug–target interaction, heterogeneous network, adaptive meta-graph, graph convolutional networks

## Abstract

Prediction of drug–target interactions (DTIs) is essential in medicine field, since it benefits the identification of molecular structures potentially interacting with drugs and facilitates the discovery and reposition of drugs. Recently, much attention has been attracted to network representation learning to learn rich information from heterogeneous data. Although network representation learning algorithms have achieved success in predicting DTI, several manually designed meta-graphs limit the capability of extracting complex semantic information. To address the problem, we introduce an adaptive meta-graph-based method, termed AMGDTI, for DTI prediction. In the proposed AMGDTI, the semantic information is automatically aggregated from a heterogeneous network by training an adaptive meta-graph, thereby achieving efficient information integration without requiring domain knowledge. The effectiveness of the proposed AMGDTI is verified on two benchmark datasets. Experimental results demonstrate that the AMGDTI method overall outperforms eight state-of-the-art methods in predicting DTI and achieves the accurate identification of novel DTIs. It is also verified that the adaptive meta-graph exhibits flexibility and effectively captures complex fine-grained semantic information, enabling the learning of intricate heterogeneous network topology and the inference of potential drug–target relationship.

## INTRODUCTION

The interactions between drugs and targets play a critical role in the process of drug discovery. The successful prediction of interactions between drugs and targets helps to identify new drug compounds that can bind to specific biological targets, which facilitates drug discovery, repositioning and the prediction of drug side effects [1]. Although laboratory experiments, utilizing various techniques of classical and reverse pharmacology, can be used to infer drug–target interactions (DTIs), they are both time-consuming and expensive [2]. Therefore, there is a growing need for in-silico prediction of DTIs, which can effectively reduce the search space for laboratory experiments while achieving high accuracy in predicting possible interactions.

There are two main computational tasks for predicting DTIs, i.e. DTI probability prediction and drug–target binding affinity prediction. Specifically, the DTI probability prediction task was traditionally modeled as a binary classification problem which predicts potential links between drugs and targets. The drug–target binding affinity prediction task focuses on an important piece of information about protein–ligand interactions (i.e. the binding affinity values), and therefore, the drug–target binding affinity prediction task is always considered as a regression task [3]. In our work, we mainly focus on the DTI probability prediction task.

The existing mainstream DTI prediction methods are machine learning-based approaches, which can be further categorized into three categories: feature-based methods, similarity-based methods and network-based methods. Feature-based methods transform drug and target data into feature vectors by using effective feature extraction and processing methods to describe drug and target information for DTI prediction. For example, Tabei *et al.* [4] utilized a tensor product approach to combine 881 drug compound structures with 876 protein domain structural features of target proteins. Ru *et al.* [5] employed a distance-based top-n-gram algorithm and general descriptors of compounds to extract protein and drug features. DeepDTI first automatically extracted drug and target features from the simple chemical substructure and sequence information and then constructed classification models using deep belief networks to exploit potential DTIs [6]. However, lacking of explicit features may cause difficulties in accurately predicting DTIs. Similarity-based methods assume that similar drugs usually interact with the same target. For instance, NRLMF calculated the probability of DTI exploiting the drug similarities and target similarities through logistic matrix factorization [7]. Chen *et al.* [8] proposed a model named NRWRH based on the integration of drug–drug, protein–protein and DTI networks. TransformerCPI improved the accuracy of drug–protein interaction prediction through sequence-based deep learning and self-attention mechanisms [9].

Network-based methods infer potential edges from known heterogeneous network, where nodes represent drugs, targets, side effects and other entities related with drugs or targets. For instance, Ezzat *et al.* [10] utilized a matrix factorization approach with graph regularization to predict DTIs based on a drug–target bipartite graphs. With the increment of data associated with drugs/targets, advanced network-based approaches are successively proposed based on complex heterogeneous networks and achieved satisfactory performance on DTIs prediction. Yan *et al.* [11] proposed a network-based label propagation method with mutual interaction information derived from heterogeneous network to infer potential DTIs. DTINet employed random walk with restart and diffusion component analysis to obtain features of drugs and proteins; then, inductive matrix complementation was used to distinguish DTIs [12]. DDR applied a random forest model to recognize DTIs using various graph-based features [13]. MKLC-BiRW applied multiple kernel learning and clustering methods to integrate heterogeneous information sources, and the bi-random walk was used to infer potential DTIs [14]. MultiDTI employed convolutional neural networks to ascertain sequence features and predicted DTIs based on nodes’ distance in a shared space [15]. However, these network-based models have a limited ability to learn the complex structure of the network due to the multi-source nature of the heterogeneous network. More recently, graph neural network (GNN) models have demonstrated their efficacy in capturing the intricate topological structure of heterogeneous networks. NeoDTI utilized GNN to learn topology-preserving node features through multiple messaging and aggregation [16]. GCN-DTI applied a graph convolutional network (GCN) to discern features of each drug–protein pair, which subsequently serve as input, followed by deploying deep neural networks for DTI prediction [17]. EEG-DTI leveraged a GCN to extract features from eight biological networks, computing DTI scores via the inner product method based on the derived low-dimensional representations [18]. Nowadays, one of the fundamental challenges for GNN-based models is how to effectively learn the embedding of nodes and edges in heterogeneous network.

Recently, meta-paths serve as effective strategies for GNN-based DTI prediction models to optimally discern neighbors to aggregate the information of nodes and edges in heterogeneous networks. Specifically, the meta-paths in biological heterogeneous networks may include specific metabolic pathways or biological principles, which benefit for the interpretation of DTI prediction [19]. For instance, IMCHGAN adopted a graph attention network with a meta-path level attention mechanism to learn the drug and target embeddings for inferring potential DTI [20]. Tanvir *et al.* [21] employed meta-paths to extract rich semantic relationships between entities, and feeded a comprehensive feature to classifiers for drug-drug interaction prediction. These meta-paths are always designed empirically, which rely heavily on domain knowledge and are hardly transferred to other heterogeneous networks [22]. Furthermore, although heterogeneous networks include multiple information, not all information are useful for DTI prediction. Recent advances of network architecture search technologies suggest that it is possible to develop an adaptive network architecture for DTI prediction. HampDTI designed a trainable meta-path based on the network architecture search technology and learned low-dimensional features of drugs and targets by using the generated meta-path graph [19]. Compared with trainable meta-paths, expressive meta-graphs possess a superior capacity to capture complex semantic information [23].

In this article, an **A**daptive **M**eta-**G**raph-based **D**rug–**T**arget **I**nteraction prediction approach, named ‘AMGDTI’, is proposed to predict potential DTIs on a heterogeneous network. AMGDTI automatically searches for an adaptive meta-graph from a heterogeneous network without requiring domain knowledge, where the adaptive meta-graph exhibits a more flexible structure and enables an efficient integration of complex multiple semantic relationships and structures information embedded in the heterogeneous network, which is the key to achieving a satisfactory result. Specifically, the main contributions of this paper are summarized as follows.

(1) An adaptive meta-graph searching strategy is proposed in AMGDTI, which considers automatically searching for an efficient information integration way without domain knowledge. The adaptive meta-graph exhibits a more flexible structure and well represent fine-grained complex semantic messages, which is utilized to learn the complex topology of heterogeneous networks and infer the potential relationships between drugs and targets. Although there are several works reported for DTI prediction by using meta-paths, but little work focuses on aggregating information based on adaptive meta-graphs.

(2) Based on the adaptive meta-graph searching strategy, a DTI prediction method, named AMGDTI, is proposed based on heterogeneous networks. The effectiveness of the proposed AMGDTI is verified on two benchmark datasets. Experimental results demonstrate that our approach overall outperforms eight state-of-the-art methods in predicting DTI, establishing AMGDTI as a competitive and promising solution. Despite this, multi-omics data, such as transcriptome, may enhance the performance on complex DTIs prediction task.

## MATERIALS AND METHODS

### Heterogeneous networks

The performance of AMGDTI is evaluated on two benchmark heterogeneous networks, named heterogeneous network $N_A$ and $N_B$, which are constructed using Luo’s dataset [12] and Zheng’s dataset [24], respectively. Luo’s dataset includes four kinds of objects (drugs, target proteins, diseases and side effects) as well as several interactions between them as shown in Table 1. These objects and interactions are integrated to construct the heterogeneous network $N_A$. Compared with $N_A$, the heterogeneous network $N_B$ contains more attributes of drugs and proteins as additional objects, such as fundamental chemical substructures, basic drug substituents, GO terms and so on. In addition, the numbers of drugs and proteins in $N_B$ are lager than those in $N_A$, as shown in Table 2.

**Table 1:** The information of nodes and edges in the heterogeneous network $N_A$

Node type	Num	Edge type	Num
Drug	708	Drug–drug (interaction)	1 0036
Protein	1512	Drug–drug (similarity)	50 1264
Disease	5603	Drug–protein	1923
Side effect	4192	Drug–disease	19 9214
		Drug–side effect	8 0164
		Protein–disease	1 59 6745
		Protein–protein (interaction)	7363
		Protein–protein (similarity)	2 28 6144

**Table 2:** The information of nodes and edges in the heterogeneous network $N_B$

Node type	Num	Edge type	Num
Drug	1094	Drug–drug	1 19 6836
Protein	1556	Drug–protein	1 1819
Chemical structure	881	Drug–chemical substructure	13 3880
Side effect	4063	Drug–side effect	12 2792
Substituent	738	Drug–substituent	2 0798
GO term	4098	Protein–GO term	3 5980
		Protein–protein	2 42 1136

### AMGDTI model

**Figure 1:**
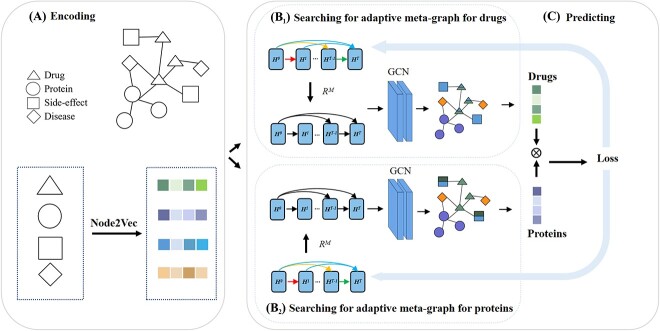
Overview of the AMGDTI algorithm, which is divided into three steps. (**A**) Constructing the heterogeneous network with multi-source biomedical data and employing the Node2Vec algorithm to encode the node representation. (**B**) Searching for the adaptive meta-graph for the information aggregation of drugs ($b_1$) and protein targets ($b_2$) based on GCN in the heterogeneous network, respectively. (**C**) Utilizing the inner product of the aggregated feature representation of drugs and proteins to predict potential DTI.

This section describes the details of the proposed AMGDTI model to predict potential drug–protein interactions. The flowchart of AMGDTI is shown in Figure 1.

#### Encoding the node representation

In our work, Node2Vec [25] is employed to encode nodes in a heterogeneous network, since Node2Vec is widely used to convert nodes into low-dimensional vectors as initial features before graph convolution operations (Figure 1A). Node2Vec performs multiple random walks to obtain sequences of nodes. Subsequently, utilizing the skip-gram model from Word2Vec [26], the sequences of nodes obtained from the random walks are used as training samples to generate low-dimensional embedding vectors for the nodes. This approach effectively captures both the local neighborhood and global structural information between nodes and subsequently encodes it into vector representations.

#### Adaptive meta-graph

In what follows, we describe the definition of an adaptive meta-graph, and the way to aggregate semantic information guided by adaptive meta-graphs within a heterogeneous network.

**Figure 2:**
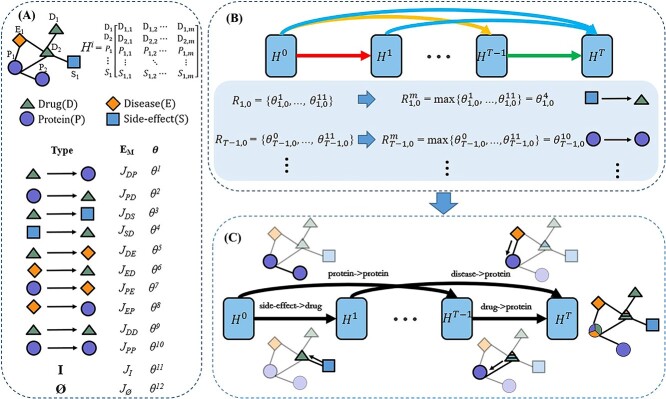
Illustration of adaptive meta-graph construction and information aggregation. (**A**) Representation of nodes and edges in an adaptive meta-graph. $J_*$ represents the edge types of the heterogeneous graph. $D$ stands for Drug, $P$ stands for Protein, $E$ stands for diseasE and $S$ stands for Side-effect, $I$ signifies participation in the composition process but without information aggregation, while $\emptyset $ denotes a lack of participation in the composition process. For example, $J_{DP}$ represents the transmission of node information from the drug to the protein, while $J_{PD}$ represents the transmission of node information from the protein to the drug. $\theta ^{*}$ represent the normalized parameters of the corresponding edges, which are used to quantify the importance of the edges. (**B**) The process of selecting the optimal link type of information propagation for an adaptive meta-graph (with the example of information propagation for protein target). (**C**) The process of information propagation based on the constructed adaptive meta-graph.

**Definition of an adaptive meta-graph.** An adaptive meta-graph is formally defined as a directed acyclic graph $M = (V_{M}, E_{M})$, where $V_{M}=\{H^0,H^1,\cdots ,H^T\}$ refers to the collection of a heterogeneous network’s node-feature $H^{i}$ within the $i$th information propagation ($i\in \{0,1,\cdots ,T\}$, $T$ denotes the number of aggregation iterations in the heterogeneous network). For simplicity, the node-feature of a heterogeneous network within the $i$th information propagation is called the $i$th state of the heterogeneous network. The set of directed links, $E_{M}$, represents the collection of information propagation modes. For example, if the directed link from $H^{0}$ to $H^{1}$ is labeled as ‘side-effect $\rightarrow $ drug’ (Figure 2C), then it means $H^1$ is achieved by aggregating the features of ‘side-effect’ nodes to those of ‘drug’ nodes in $H^0$. In the proposed adaptive meta-graph, any of the previous $t$ states ($H^{0},H^{1},\cdots , H^{t-1}$) can affect the current state $H^{t}$ by a certain information propagation mode, thereby generating the skip structures between different states of a heterogeneous network. Thus, the first feature of the proposed meta-graph is the skip structure between nodes, which enables more effective extraction of complex semantic information from a heterogeneous network.

Another feature of the proposed adaptive meta-graph is that each link in a meta-graph is adaptively determined. That is, whether a previous state of a heterogeneous network affects the current state, the way to affect the current state is determined adaptively. To this end, all the edge types in a heterogeneous network are served as the possible information propagation modes. Besides, another two information propagation modes ($J_I$ and $J_{\emptyset }$) are added, where the propagation mode $J_I$ means that the current state is equal to a previous state, and the mode $J_{\emptyset }$ denotes that a previous state could not affect the current state. Specifically, in AMGDTI, there are $12$ kinds of possible links between nodes, i.e. $E_M=\{J_{DP}, J_{PD}, J_{DS}, J_{SD}, J_{DE}, J_{ED}, J_{PE}, J_{EP}, J_{DD}, J_{PP}, J_I, J_{\emptyset }\}$, where the first 10 types of links are corresponding to the edge types in the heterogeneous graph, and $J_{\emptyset }$, $J_I$ are newly designed to make the adaptive meta-graph more flexible. The way to adaptively select the information propagation modes among different states will be illustrated in the following.

**Construction of an adaptive meta-graph.** For the DTI prediction, an adaptive meta-graph is applied to guide the information aggregation in a heterogeneous network to obtain the features of both drugs and proteins. In what follows, we illustrate the way to construct an adaptive meta-graph. Firstly, the number of nodes in an adaptive meta-graph depends on the times of information propagation in the heterogeneous network. Suppose that the feature of nodes propagates $T$ times in the heterogeneous network. Then, the nodes in the adaptive meta-graph are $V_m=\{H^0,H^1,\cdots , H^T\}$.

Next, the possible connections between a pair of nodes are chosen from $E_M$. To be specific, given two nodes, $H^i$ and $H^t$, where $0 \leq{i,t} \leq{T}$, $i\in{N}$ and $t\in{N}$. The proposed method checks whether $H^t$ is the next state of $H^i$ and whether $H^t$ is the last state. If $i=t-1$ and $t<T$, then the possible connections from $H^{i}$ to $H^t$ are those in $E_M$ except for $J_{\emptyset }$, since the node-feature in the $i$th information propagation affects that of in the $t$th propagation by a certain mode. That is, the possible connections from $H^i$ to $H^t$ are the elements in the set $R_{t, i}=E_M-\{J_{\emptyset }\}$, if $i=t-1$ and $t<T$ (Eq. 1). If $t<T$ but $i<t-1$, then the state $H^i$ may not affect the state $H^t$. In this situation, the possible connections from $H^i$ to $H^t$ include $J_{\emptyset }$, i.e. $R_{t, i}=E_M$. Moreover, if $H^t$ is the last state ($t=T$), then the possible connections from $H^{t-1}$ to $H^t$ are further restrict to those connections related with either drugs or targets. On one hand, to achieve the node-feature of drugs, the possible connections with constraint ($C^{\prime}$) are chosen as the types with the form of ‘$*\rightarrow $drug’. On the other hand, we choose those related with proteins with the form of ‘$*\rightarrow $protein’ to update the node-feature of proteins. For example, on the heterogeneous network $N_A$, four modes are chosen to update the node-feature of drugs, i.e. ‘drug $\rightarrow $ drug ’, ‘protein $\rightarrow $ drug’, ‘disease $\rightarrow $ drug’ and ‘side-effect $\rightarrow $ drug’. That is, in this situation, $C^{\prime}=\{J_{DD},J_{PD},J_{ED},J_{SD}\}$. Compared with the possible connections under the situation of $i=t-1$ and $t=T$, those under the situation of $i<t-1$ and $t=T$ add two types of connections, i.e. $J_I$ and $J_{\emptyset }$. The possible connections from $H^i$ to $H^t$ in four situations are listed as follows: 


(1) \begin{align*}& R_{t, i}=\left\{\begin{array}{@{}ll} E_M-\{J_{\emptyset}\}, & i=t-1, t<T\\ E_M, & i<t-1, t<T\\ C^{\prime}, & i=t-1, t=T\\ C^{\prime}\cup J_I \cup J_{\emptyset}, & i<t-1, t=T\\ \end{array}\right.\kern-6pt,\end{align*}

Thirdly, a connection from $H^i$ to $H^t$ is adaptively chosen from all possible connections. In the proposed AMGDT, a parameter $\theta _{t,i}^{*}$ is assigned to each possible link from $H^i$ to $H^t$ to represent the possibility of the link to be selected. For example, the possibility of the connection ‘drug $\rightarrow $ protein’ ($J_{DP}$) from $H^0$ to $H^2$ is assigned a parameter $\theta _{0,2}^{1}$. The connection with the maximum value $\theta _{t,i}^{m}=max(\theta _{t,i}^{0}, \ldots , \theta _{t,i}^{11})$ will have a large probability to be the link from $H^i$ to $H^t$. Besides, in order to increase the diversity of possible meta-graphs, the link from $H^i$ to $H^t$ is randomly chosen from $R_{t,i}$. In the proposed AMGDTI, the final type of links from $H^i$ to $H^t$ is randomly chosen from $R_{t,i}$ with the possibility $p_i$, and with the possibility of $1-p_i$ to be that with the maximum value of the parameter $\theta $. The parameter $p_{i}\in (0,1)$ is set to be a small value that promotes the exploration of various message passing options in the initial stages, gradually reducing to $0$ as the value of $i$ increases. Formally, the type of connections from the node $H^i$ to $H^t$ in the adaptive meta-graph can be determined as follows: 


(2) \begin{align*}& R_{t, i}^{m}=\left\{\begin{array}{@{}ll} {\theta_{t,i}^{m}} &m=\arg \max _{\mathrm{n}} \theta_{\mathrm{t}, \mathrm{i}}^{n}\ \text{with probability}\ 1-p_{i} \\ \operatorname{rand}\left(R_{t, i}\right) & \text{with probability}\ p_{i} \end{array}\right.\kern-6pt,\end{align*}

where ‘rand($\cdot $)’ denotes the random and uniform sampling of an element from a given set.

The calculation of $\theta _{t,i}^{m}$ is the key to adaptively choose the type of links. Here, a network architecture search-based method (DiffMG [27]) is used to measure the value of $\theta _{t,i}^{m}$. To be specific, the significance of each link is firstly initialized as a random number from $[0,1]$. For example, the significance of the $n$th type of links from $H^i$ to $H^t$ is initialized to be $0.3$, i.e. $\alpha _{t, i}^{n}=0.3$. According to Eq. 2, the meta-graph is initialized. The node feature in a heterogeneous network is updated guided by the initialized adaptive meta-graph. The detail of the information aggregation is illustrated in the next subsection. Then, the significance of each link is updated by optimizing two objectives, where the two optimization objectives are the validation loss $L_{val}$ and the training loss $L_{tra}$, respectively. Suppose $L$ is the loss function, and $\omega $ represents the network structure parameter. The significance of each link is adaptively optimized by Eq. 3


(3) \begin{align*} \min _{\alpha} {{L}_{\text{val }}}(\boldsymbol{\omega}^{*}(\alpha), \alpha), \text{ s.t. } \boldsymbol{\omega}^{*}(\alpha)=\arg \min _{\boldsymbol{\omega}} {{L}}_{\text{tra }}(\boldsymbol{\omega}, \alpha) \end{align*}

(4) \begin{align*} L=-\sum_{(d, p) \in \Omega^{+}}\log \sigma\left(h_{d}^{T} h_{p}\right)-\sum_{\left(d^{\prime}, p^{\prime}\right) \in \Omega^{-}} \log \sigma\left(-h_{\mathrm{d}^{\prime}}^{T} h_{p^{\prime}}\right), \end{align*}

where $\Omega ^{+} $ is the set of known drug–protein interactions (positive samples), and $\Omega ^{-} $ is the negative samples which contain the drugs and proteins without interactions. $h_{d}$, $h_{p}$ are, respectively, the node representations of drugs and proteins obtained from positive samples, while $h_{d^{\prime}}$, $h_{p^{\prime}}$ are those achieved from negative samples. $log\sigma$ denotes the logsigmoid function. Furthermore, the possibility of the $n$th type of links from $H^i$ to $H^t$, denoted by $\theta _{t,i}^{n}$, is achieved by normalizing the significance of the link ($\alpha _{t,i}^{n}$) which is as follows: 


(5) \begin{align*}& \theta_{t, i}^{n}=\frac{\exp \left(\alpha_{t, i}^{n}\right)}{\sum_{n^{\prime}=0}^{\left|R_{t, i}\right|} \exp \left(\alpha_{t, i}^{n^{\prime}}\right)}.\end{align*}

#### Information aggregation guided by adaptive meta-graphs

Given an adaptive meta-graph and the first $(t-1)$th state of a heterogeneous network, the $t$th state of a heterogeneous network is updated by aggregating information based on GCN (Figure 2C). Specifically, the message propagation for $H^{t}$ in adaptation meta-graphs is delineated as follows: 


(6) \begin{align*}& H^t=gelu\left[\sum_{i=0}^{t-1}{g_{t,i}(H^i,R_{t,i}^m)}\right],\end{align*}

where $g_{t,i}$ denotes message propagation using the GCN model, and subscripe represents the set of edge types from the $i$th intermediate state $H^{i} $ to the $t$th state. $gelu(\cdot)$ indicates GELU activation function. $g_{t,i}(H^{i}, R_{t, i}^{m})$ denotes the propagation of information from the $i$th state along an edge of type $R_{t, i}^{m}$ to $t$th state. $g_{t,i}$ is delineated as follows: 


(7) \begin{align*}& g_{t, i}\left(H^{i}, R_{t, i}^{m}\right)=\tilde{D}_{t, i}^{-\frac{1}{2}} \tilde{R}_{t, i}^{m} \tilde{D}_{t, i}^{-\frac{1}{2}} H^{i} W_{t,i},\end{align*}

where $\tilde{R}_{t, i}^{m}=R_{t, i}^{m}+I_{t, i}$, $I_{t, i}$ is an identity matrix, $\tilde{D}_{t, i}$ is the diagonal degree matrix of $\tilde{R}_{t, i}^{m}$ and $W_{t, i}$ is the weight matrix of GCN. The states of each node of the adaptive meta-graph are obtained sequentially according to the above process, and the $T$th state $H^T$ is finally obtained.

#### DTI prediction

After performing the information aggregation process, we obtain the feature vectors of the drug and the protein and use the inner product of the two to predict the potential DTI. Given a specific drug $d$ and a particular target $p$, the interaction score $P^{dp} $ between $d$ and $p$ can be calculated as follows: 


(8) \begin{align*}& P^{d p}=\sigma\left(h_{d}^{T} h_{p}\right),\end{align*}

where $h_{d} $ and $h_{p} $ are the feature representations of drugs and targets, respectively. $\sigma $ is the sigmoid function.

## RESULTS

### Baseline methods

To evaluate the performance of the proposed model, we compared AMGDTI with the six state-of-the-art drug–target prediction models as follows.

- DTINet [11] learns the low-dimensional vector representations of nodes in a constructed heterogeneous network by using a network diffusion algorithm and detects new DTIs based on a matrix completion method.
- NeoDTI [16] is the first framework to integrate the feature extraction techniques with the DTI prediction methods into an end-to-end learning framework, where the feature of a node is achieved by aggregating all of its neighborhood information.
- GCN-DTI [17] learns the features for each node in a constructed drug–protein pair network by using a GCN and then uses a deep neural network to predict DTIs.
- IMCHGAN [20] is a meta-path-based DTIs prediction model, where the drug and target embeddings are learned by adopting a graph attention network with meta-path level attention mechanism.
- EEG-DTI [18] is a heterogeneous GCNs-based framework for the prediction of DTIs, where the feature representation of each node is generated by aggregating the features of its neighbors connecting by different types of edges in each layer of the GCN.
- HampDTI [19] developed a meta-path graph structure that indirectly determines the importance of each possible meta-path connecting a drug and a target. Following this, GCNs are employed on the resulting meta-path graph to learn reduced-dimension drug and target attributes for DTI prediction.
- DeepConv-DTI [28] uses convolutional filters to capture local residue patterns participating in DTIs, uses data as high-level input, constructs model protein features and concatenates drug features. Finally, the DTIs probability is predicted through the fully connected layer.
- TripletMultiDTI [29] employs a combination of triplet loss and task prediction loss to create a more discriminative feature representation of drug–target pairs, leading to improved prediction performance by enhancing the clustering of feature space for similar drug–target pairs and distinguishing dissimilar ones.

DeepConv-DTI is a sequence-based method, while others are network-based methods. Network-based methods execute the similar procedures with the proposed model, i.e. they firstly learn the feature representations of nodes in a heterogeneous network and then predict DTIs.

### Parameter setting

The proposed AMGDTI model is implemented on the PyTorch framework with the Adam optimizer [30], where the learning rate is $6e-3$, the weight decay rate is $1e-3$, the hidden size is $64$, the decay rate is $0.2$ and $150$ epochs are used to train. For Node2Vec, we set the walk size $ws=100$, the number of walks $nw=15$, the degree of forward movement $p=1$ and the degree of backward movement $q=1$. Besides, the parameters of the above-mentioned baseline methods follow the settings in their papers.

### Performance evaluation

To evaluate the performance of AMGDTI, we perform 5-fold cross-validation in two benchmark datasets. Since the number of unknown DTIs is much larger than that of known drug–target pairs in each of the two benchmark datasets, unknown DTIs are under-sampled to make the size the same as the number of known DTIs, resulting the positive samples (i.e. all of the known DTIs) and the negative samples (i.e. the unknown DTIs selected according to above principle). For the 5-fold cross-validation, we randomly select $60\%$ of positive samples and $60\%$ the negative samples to train the model. Besides, randomly selected $20\%$ of positive samples and $20\%$ of negative samples are used as the validation set to tune the parameters. The remaining $20\%$ of positive samples and negative samples are selected as the testing set. The area under the receiver operating characteristic curve (AUC) and the area under the precision-recall curve (AUPRC) are used to evaluate the performance of the proposed AMGDTI, since they have been widely used in the research of DTI prediction. In our work, $5$ times of 5-fold cross-validation are perfomed on two benchmark datasets, and the best results are calculated to show the performance of each method. As shown in Table 3, the following two observations can be obtained.

**Table 3:** The Comparison of AUC, AUPRC, $P$-value and Params between various models

Model	Network $N_A$			Network $N_B$			Params
	AUC	AUPRC	$P$ -value	AUC	AUPRC	$P$ -value	
DTINet	0.879	0.906	5.31e−10	0.889	0.900	2.08e−7	*
NeoDTI	0.955	0.889	4.84e−8	0.946	0.846	1.99e−4	9.98e10
GCN-DTI	0.918	0.897	1.76e−8	0.922	0.914	1.61e−4	*
IMCHGAN	0.956	0.903	1.06e−4	0.946	0.929	1.35e−3	4.44e5
EEG-DTI	0.954	0.964	2.24e−5	0.942	0.941	2.41e−4	1.84e6
HampDTI	0.928	0.927	5.51e−9	–	–	–	9.92e4
DeepConv-DTI	0.909	0.917	3.23e−8	–	–	–	1.52e6
TripletMultiDTI	**0.991**	**0.990**	6.53e−4	–	–	–	1.53e7
AMGDTI	0.977	0.977		**0.973**	**0.971**		1.33e5

‘-’ indicates that the model is not applicable to this dataset. ‘$P$-value’ is calculated using AUC as a statistic. ‘*’ indicates that the model is not convenient for parameter statistics. ‘Params’ represents the parameter quantity of the model.

 First, the proposed adaptive meta-graph based AMGDTI achievesan overall superior performance over two datasets. For example, on the Network N_B, AMGDTI shows the best performance, and the meta-path-based method IMCHGAN ranks the second. The performances of other methods considered here are not good as above two methods, due to the fact that they aggregate node information without distinguishing edge types (except HampDTI). It is demonstrated that different types of edges in heterogeneous networks play different roles in aggregating node information. Both IMCHGAN and HampDTI enable the automatic learning of latent feature representations from bioinformatics networks, which avoids the need for domain-specific knowledge. The IMCHGAN algorithm constructs corresponding meta-paths for drugs and protein targets, extracting potential feature representations for drugs and target proteins separately. In contrast, HampDTI designs a single meta-path for the heterogeneous network to predict DTI. We consider that due to differences in effectively extracting potential features for drugs and targets, the separate design of meta-path/meta-graph in the context of heterogeneous network modeling for DTI prediction should outperform the sole design of a single meta-path/meta-graph. As for AMGDTI, it constructs meta-graphs for drugs and protein targets to extract potential features, respectively.Moreover, the adaptive meta-graph in AMGDTI enables the flexible extraction of refined semantic features. Therefore, although AMGDTI, IMCHGAN, and HampDTI are all algorithms designed for automatic meta-path/meta-graph to predict DTI, AMGDTI performs the best in terms of prediction performance.Second, the proposed adaptive meta-graph is more suit to aggregating node information for drug-target interaction prediction. Specificly, the AUC obtained by AMGDTI is $0.977$ and $0.973$ on Luo’s dataset and Zheng’s dataset, respetively, which is $2.1\%$ and $2.5\%$ higher than those obtained by IMCHGAN. The promising performance of the proposed method AMGDTI may partly be due to the introduce of an adaptive meta-graph module, since AMGDTI discerns more useful edge types for predicting drug–target intersections. HampDTI relies on both the SMILES sequences of the drug and the amino acid sequences of the target. However, Zheng’s dataset lacks the necessary sequence information.

**Figure 3:**
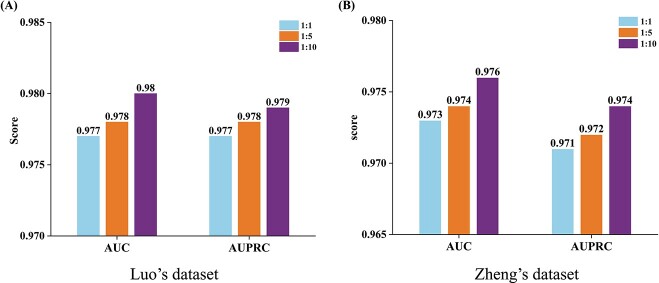
Comparison of different positive and negative sample ratios on Luo and Zheng’s dataset.

In our work, DTI pairs with known interactions are considered as positive samples and the remaining drug–target pairs as negative samples. Due to the inherent imbalance in the number of positive and negative samples, the number of positive samples is significantly lower than that of negative samples. To evaluate whether the number of positive and negative samples affects the performance of the proposed AMGDTI, we conducted experiments employing varying ratios of positive and negative samples. Figure 3 presents the AUC and AUPRC values of AMGDTI when the positive and negative sample ratios are set to be $1:1$, $1:5$ and $1:10$. From the figure, it can be found that the performance of AMGDTI differs little as the positive and negative sample ratios changes.

### Effectiveness of adaptive meta-graph

**Figure 4:**
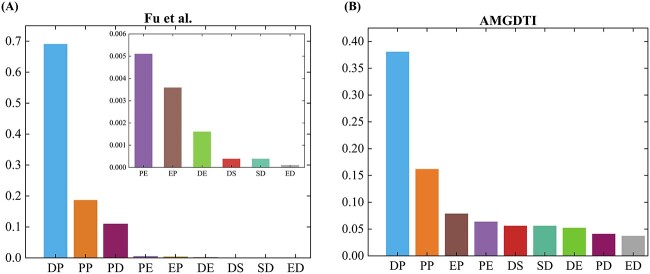
Comparison of the frequency of edge types in meta-graphs. (**A**) The frequency of edge types in meta-paths determined by the method proposed by Fu *et al.* (**B**) The frequency of edge types in adaptive meta-graphs determined by AMGDTI.

Different meta-graphs influence the DTI prediction results. A key structure of meta-graphs is the type of edges. To explore which kind of information aggregation modes largely affects the prediction of DTI, Figure 4 shows the frequency of edge types in meta-graphs achieved by the proposed AMGDTI and the method proposed by Fu *et al.* [31] in the heterogeneous network $N_A$, respectively. Specifically, in the method proposed by Fu *et al.*, $51$ meta-paths with lengths ranging from $2$ to $4$ are manually enumerated, and the edge type (‘drug–target’) contributes the most to predicting DTI, followed by ‘target–target’ (Figure 4(A)). Similarly, in AMGDTI, we randomly selected negative samples and chose $T$ to be either $3$ or $4$ for each trial and achieved $51$ adaptive meta-graphs. From Figure 4(B), it is found that the two most frequent edge types are also the ‘drug–target’ and ‘target–target’, which are the same as those achieved by the method proposed by Hu *et al.* The other types of interactions play a supporting role in predicting DTI. Besides, We provided a $t$-test on DTI prediction results to further investigate the difference among various methods. The $P$-value generated by considered models on two datasets are also listed in Table 3. Form above results, it is indicated that the proposed AMGDTI can effectively detect useful information aggregation modes for DTI prediction.

**Figure 5:**
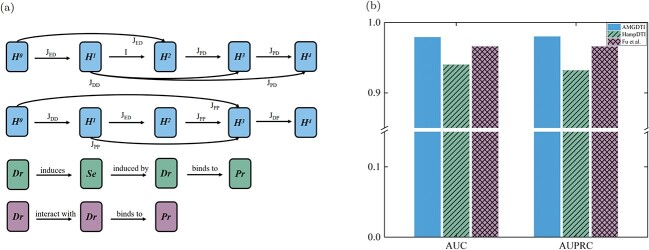
Meta-graphs and their effects on DTI prediction. (**A**) Meta-graphs achieved by three methods include the best adaptive meta-graph obtained by AMGDTI (the first for Drugs and the second for Proteins), the optimal meta-path acquired by HampDTI (the third figure), and the method proposed by Fu et al. (the fourth figure). (**B**) Comparison of DTI prediction results by using the meta-graphs achieved by AMGDTI, HampDTI and the method proposed by Fu *et al.* on the heterogeneous network $N_A$.

To further show the effectiveness of the proposed adaptive meta-graph, Figure 5 shows the comparison of the prediction results by using the best meta-paths achieved by HampDTI and the method proposed by Fu *et al.*, as well as the optimal adaptive meta-graph achieved by AMGDTI. To be specific, above three meta-graphs were, respectively, used to aggregate information on the heterogeneous network $N_A$, while the way to calculate the interaction score was the same. Besides, 5-fold cross-validation was performed, and AUC and AUPRC were used as the evaluation indicators. From Figure 5B, it can be seen that the AUC and AUPRC achieved by the proposed optimal adaptive meta-graph are higher than those achieved by the other two best meta-graphs, indicating that the proposed optimal adaptive meta-graph can effectively aggregate information in a heterogeneous network, which is benefit for DTI prediction.

### Ablation study

The proposed AMGDTI mainly contains three essential steps, i.e. encoding the nodes in a heterogeneous network by Node2vec, the construction of adaptive meta-graphs and the information aggregation guided by adaptive meta-graphs, where the strategy of selecting possible links with constraints is the key to construct adaptive meta-graphs. Here, the ablation experiments were considered to check the contribution of these key components. The model variants are summarized as follows: (1) AMGDTI-Node2vec means AMGDTI without Node2vec but with one-hot encoding strategy; (2)AMGDTI-Constraints denotes AMGDTI without the strategy of selecting possible links with constraints; and (3) AMGDTI-AMP means that the adaptive meta-graph in AMGDTI is replaced by a manually designed meta-path (i.e. the best meta-path achieved by HampDTI [19]).

Table 4 presents the AUC and AUPRC values of the proposed AMGDTI, and the three variants on two heterogeneous networks. From the table, we can find that the performance of AMGDTI is better than other three variants. This result indicates that the integration of three essential steps helps to improve the prediction performance of AMGDTI. Besides, the results suggest the effectiveness of Node2vec in node encoding, the strategy of selecting possible links with constraints and the information aggregation guided by adaptive meta-graphs. In addition, we found that AMGDTI-AMP shows the maximum performance degradation. This finding indicates that the information aggregation guided by adaptive meta-graphs is the key to achieving a satisfactory result, and adaptive meta-graphs can well represent fine-grained complex semantic messages, and benefit to learn the complex topology of heterogeneous networks and infer potential relationships between drugs and targets.

**Table 4:** Performance of AMGDTI and three variants on heterogeneous networks

Method	Network $N_A$		Network $N_B$	
	AUC	AUPRC	AUC	AUPRC
AMGDTI-Node2vec	0.963	0.951	0.936	0.921
AMGDTI-Constraints	0.965	0.953	0.966	0.954
AMGDTI-AMP	0.958	0.956	–	-
AMGDTI	**0.977**	**0.977**	**0.973**	**0.971**

‘-’ indicates that the model is not applicable to the dataset.

### Prediction of potential DTIs

Potential DTI is predicted based on the constructed heterogeneous network and the AMGDTI model. Utilizing heterogeneous network $N_A$ containing drugs, targets, side effects and diseases, the AMGDTI model is trained for the prediction of potential DTI using an equal number of positive and negative samples. Utilizing the trained AMGDTI model, predictions are made for all unconfirmed drug–target relationship pairs, and the top 10 highest scoring potential DTI results are presented in Table 5.

**Table 5:** The prediction and validation of novel (potential) DTIs

Rank	Drug ID	Drug Name	Target ID	Target Name	Evidence
1	DB00502	Haloperidol	P08172	CHRM2	DrugBank5.0 (DB00334),KEGG (hsa04080)
2	DB01136	Carvedilol	P35368	ADRA1B	DrugBank5.0 (Proved)
3	DB01280	Nelarabine	Q02880	TOP2B	Unknown
4	DB00418	Secobarbital	P47870	GABRB2	DrugBank5.0 (DB06716), KEGG (hsa04080)
5	DB00398	Sorafenib	Q08345	DDR1	Unknown
6	DB01236	Sevoflurane	O60391	GRIN3B	DrugBank5.0 (DB01520), KEGG (hsa04080)
7	DB00734	Risperidone	P08173	CHRM4	DrugBank5.0 (DB09167), KEGG (hsa04080)
8	DB00370	Mirtazapine	Q9NYX4	CALY	DrugBank5.0 (DB00370), KEGG (hsa04080)
9	DB01159	Halothane	P18505	GABRB1	DrugBank5.0 (Proved)
10	DB00449	Dipivefrin	P25100	CHRM2	KEGG (Proved)

In order to ascertain the credibility of the predicted potential DTIs, various reference databases are consulted to seek corroborative evidence, such as KEGG and DrugBank version 5.0 [32]. DrugBank database reports the relevant drugs for the target and the relevant targets for the drug, and Drugbank 5.0 is the latest version, documenting the DTI identified in recent research. KEGG database reports the relevant targets for the drug, and KEGG PATHWAY [33] stores data on protein metabolic pathways.

In our examination of the top 10 potential DTI rankings, the second-ranked interaction involving Carvedilol and ADRA1B [34], the ninth-ranked interaction between Halothane and GABRB1 [35] and the tenth-ranked interaction involving Dipivefrin and CHRM2 [36] have been substantiated by the latest database as demonstrating authentic DTIs. These findings underscore the reliability of our predictive methodology in elucidating biologically relevant DTIs.

Numerous inferred DTIs lack direct verification; however, specific biological hypotheses enable the deduction of potential DTI associations [37]. This premise relies on the notion that similar drugs typically engage with identical target proteins. Furthermore, a drug’s interaction with a protein may influence the expression of other proteins within the same pathway. For instance, the interaction prediction scores for Haloperidol and CHRM2 rank at the top. Antipsychotics Haloperidol and Olanzapine exhibit a documented interaction with CHRM2; both related to the neuroactive ligand-receptor pathway (hsa04080), suggesting a potential interaction between Haloperidol and CHRM2. Moreover, by intputing sorafenib into the model, a literature-based validation revealed that among the top 10 predicted potential targets ranked by score, three were confirmed to interact with sorafenib, encompassing FLT1 [38], CSF1R [39] and RET [40]. This substantiates the model’s robust performance in predicting targets for emerging drug entities.

## DISCUSSION AND CONCLUSION

Despite recent advances of biomedical research and technologies, DTI prediction remains a challenging task which requires the effective learning of the information of drugs and targets form a large heterogeneous network. In this study, we propose an adaptive meta-graph-based deep-learning method, AMGDTI, which automatically searches for a suitable adaptive meta-graph to predict potential DTI. AMGDTI guides the GCN in gathering neighborhood information of nodes by a novel and expressive search space, i.e. adaptive meta-graphs. From the experimental results, the enhanced performance of the proposed method mainly attributes to the adaptive meta-graph. Firstly, the adaptive meta-graph has a skip connection structure, i.e. nodes within the adaptive meta-graph (excluding the source node) possess more than one incoming link, aggregating information from multiple propagation paths. Specifically, a node in the adaptive meta-graph represents the state of the heterogeneous network after message propagation, and an edge in the adaptive meta-graph characterizes a message propagation path. Note that the adaptive meta-graph degenerates into a meta-path when there is no skip connection between nodes. The target node of the previous propagation coincides with the source node of the subsequent propagation in the adaptive meta-graph. Thus, the adaptive meta-graph exhibits a more flexible structure and can better represent fine-grained semantic messages than the meta-graph, enabling the extraction of complex semantic information. Secondly, it uncovers crucial DTI meta-paths for prediction, providing valuable insights into DTI-related research and enhances interpretability compared with prior black-box deep learning models. Thirdly, it circumvents reliance on domain knowledge, enabling the dynamic learning of adaptive meta-graphs between drugs and targets from relevant heterogeneous networks.

Furthermore, the DTIs predicted by AMGDTI could also provide potential real-world implications. An interesting finding is that Sorafenib is a potential drug for T-cell lymphoma. To be specific, Sorafenib, as a kinase inhibitor, has significant therapeutic effects in the treatment of unresectable liver cancer, advanced renal cancer and differentiated thyroid cancer. The kinase activity of DDR1 plays a central role in the development of T-cell lymphoma [41]. It is inferred that sorafenib may have potential therapeutic effects with T-cell lymphoma. Besides, Nelarabine is a purine nucleoside analog and antineoplastic agent used for the treatment of with acute T-cell lymphoblastic leukemia and T-cell lymphoblastic lymphoma. TOP2B is a DNA topoisomerase that plays an important role in maintaining genomic integrity and may also lead to chromosomal translocation and mutations, leading to acute T-cell lymphoblastic leukemia [42]. It is speculated that Nelarabine has potential therapeutic effects with acute T-cell lymphoblastic leukemia.

AMGDTI is an effective network architecture search strategy based on heterogeneous network that offers a powerful deep learning toolbox for the prediction of DTIs. If broadly applied, AMGDTI could be applied to other kinds of prediction, such as drug–drug interactions. In future endeavors, we plan to develop heterogeneous networks tailored to various disease types and incorporate multi-omics data (e.g. transcriptome, metabolome) within the disease-specific networks. The predicted DTI may prove beneficial for the treatment of specific diseases.

**Key Points**

- The prediction of drug–target interactions (DTIs) is essential in medicine field, and one of the fundamental challenges is how to effectively learn the embedding of nodes and edges in heterogeneous network. Here, we developed an adaptive meta-graph-based DTI prediction model (AMGDTI), which serves as an adaptive and efficient method for DTI prediction in a heterogeneous network.
- An adaptive meta-graph searching strategy is proposed in AMGDTI, which considers automatically searching for an efficient information integration way without domain knowledge. The adaptive meta-graph enables an efficient integration of complex multiple semantic relationships and structures information embedded in the heterogeneous network, which is the key to achieving a satisfactory result.
- The effectiveness of the proposed AMGDTI is verified on two benchmark datasets. Experimental results demonstrate that our approach overall outperforms eight state-of-the-art methods in predicting DTI. It also provides crucial meta-paths for DTI prediction, providing valuable insights into DTI-related research and enhances interpretability compared with prior black-box deep learning models.

## Supplementary Material

Supplementary_file_bbad474

## Data Availability

The datasets used in this reasarch are publicly available and can be accessed at https://github.com/ahu-bioinf-lab/AMGDTI.

## Author Biographies

**Yansen Su** is a professor at the School of Artificial Intelligence, Anhui University. She is also with Institute of Artificial Intelligence, Hefei Comprehensive National Science Center, 5089 Wangjiang West Road, 230088 Hefei, China. Her research interests include bioinformatics, deep learning and multi-objective optimization.

**Zhiyang Hu** is a master’s degree student at the School of Computer Science, Anhui University. His research interests include research of intelligent information processing and machine learning.

**Fei Wang** is currently an Assistant Professor in the Key Laboratory of Intelligent Computing and Signal Processing of Ministry of Education, School of Artificial Intelligence, Anhui University. His research interests are bioinformatics, drug repositioning, and deep learning.

**Yannan Bin** is currently an Associate Researcher in the Key Laboratory of Intelligent Computing and Signal Processing of Ministry of Education and Information Materials and Intelligent Sensing Laboratory of Anhui Province, Institutes of Physical Science and Information Technology, Anhui University. Her research is focused on bioinformatics, machine learning and bioactive peptide prediction.

**Chunhou Zheng** is currently a Professor in the Key Laboratory of Intelligent Computing and Signal Processing of Ministry of Education, School of Artificial Intelligence, Anhui University. His research interests include pattern recognition and bioinformatics.

**Haitao Li** a lecturer at the School of Artificial Intelligence, Anhui University. He is also with Information Materials and Intelligent Sensing Laboratory of Anhui Province. His main research interests include machine learning and bioinformatics.

**Haowen Chen** is an associate professor with Hunan University. His research interests include bioinformatics and artificial intelligence. He has published several research papers in these fields including Npj Systems Biology, IEEE/ACM TCBB, Methods, COMPUTERS & SECURITY etc.

**Xiangxiang Zeng** is a professor at Hunan University. His research interests include bio-computing and bioinformatics.
