# Supplementary material for: AMGDTI: drug–target interaction prediction based on adaptive meta-graph learning in heterogeneous network
Source: Brief Bioinform. 2023 Dec 25;25(1):bbad474. doi: 10.1093/bib/bbad474 (PMC10749791; doi:10.1093/bib/bbad474)
Supplement: Supplementary_file_bbad474 [file supplementary_file_bbad474.docx]

**Supplementary Information:**

**AMGDTI: Drug-target interaction prediction based on adaptive meta-graph learning in heterogeneous network**

Yansen Su,^1^ Zhiyang Hu,^2^ Fei Wang,^1^ Yannan Bin,^1^ Chunhou Zheng,^1^ Haitao Li,^1,*^ Haowen Chen^3,*^ and Xiangxiang Zeng^3^

^1^Information Materials and Intelligent Sensing Laboratory of Anhui Province, Anhui University, Hefei, 230601, China, ^2^Key Laboratory of Intelligent Computing and Signal Processing, School of Computer Science and Technology, Anhui University, Hefei, 230601, China and ^3^College of Computer Science and Electronic Engineering, Hunan University, Hunan, 410082, China

Email: liht@ahu.edu.cn; [hwchen@hnu.edu.cn](mailto:hwchen@hnu.edu.cn)

**The Supplementary Materials file includes：**

**S1. Supplementary results**

**S1.1 Experiment of redundancy**

**S1.2 Effectiveness of adaptive meta-graph**

**S1.3 Cold start analysis**

**S2. References**

**S1. Supplementary results**

**S1.1 Experiment of redundancy**

Given that the datasets may include 'redundant' DTIs, where the same protein is associated with multiple similar drugs, and vice versa, the prediction performance could be artificially boosted due to straightforward predictions in such scenarios. Therefore, we reference the DTINet algorithm's^1^ research methodology and process the redundant edges as follows: (i) remove DTIs associated with similar drugs (i.e., drug chemical structural similarity > 0.6) or similar proteins (i.e., protein sequence similarity > 40%); (ii) remove DTIs related to drugs with similar interactions (i.e., Jaccard similarity > 0.6); (iii) remove DTIs related to drugs with similar side-effects (i.e., Jaccard similarity > 0.6); (iv) remove DTIs associated with drugs or proteins having similar diseases (i.e., Jaccard similarity > 0.6). In all these test scenarios, we maintain a balanced ratio of 1:1 between positive and negative samples. The experimental results are shown in Supplementary Table 1. As expected, a

**Supplementary Table 1.** The AUC and AUPR values achieved by AMGDTI after removing redundant DTIs.

| The way to remove redundant DTIs | **AUC** | **AUPR** |
| --- | --- | --- |
| **Remove drugs or protein (structural similarity)** | 0.960 | 0.954 |
| **Remove DTI with interaction similarity** | 0.966 | 0.963 |
| **Remove DTI with side-effect similarity** | 0.976 | 0.974 |
| **Remove DTI with disease similarity** | 0.950 | 0.942 |

**S1.2 Effectiveness of adaptive meta-graph**

We have considered two experiments to analyze the sensitivity of the AMGDTI algorithm for DTI prediction: 1. Due to the incomplete nature of heterogeneous network information, we evaluate the AMGDTI algorithm performance by removing different proportions of edges from the DTI prediction; 2. Redundant data may affect the accuracy of the results, so performance of AMGDTI is evaluated after removing data redundancy by referring to paper^1^. Specifically, five times 10-fold cross-validation is performed by randomly removing 0%, 5%, 10%, 15%, and 20% of the edges constituting a new heterogeneous network to observe the results of the DTI prediction using AMGDTI.

Considering the incompleteness of the heterogeneous network, we evaluate the AMGDTI algorithm performance by removing different proportions of edges from the DTI prediction. Five times 10-fold cross-validation is performed by randomly removing 0%, 5%, 10%, 15%, and 20% of the edges constituting a new heterogeneous network to observe the results of the DTI prediction using AMGDTI. In line with our expectations, the performance decreases to a certain extent after removing a certain proportion of edges. However, we observed that the predictive performance for DTIs only decreased by approximately 1.5% as shown in Figure S1.


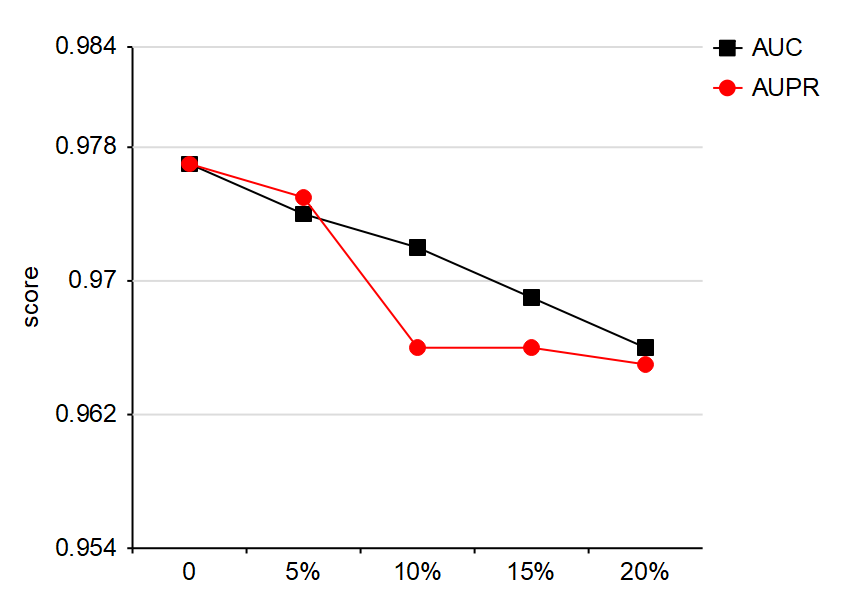


**Figure S1.** The AUC and AUPR values for DTI prediction using AMGDTI after removing edges at different proportions

We conducted a potential ablation analysis of edge types to determine which types of edges have the most significant impact on DTI prediction. Specifically, ten times 5-fold cross-validation is performed by AMGDTI for predicting DTI after removing various edge types, reflecting the impact of different information aggregation patterns on DTI prediction performance. The AUC values are shown in the Figure S2. From the figure, we observed a decrease in the predictive performance of the AMGDTI algorithm after removing a certain type of edge. However, the performance of AMGDTI in predicting DTIs varies when different types of edges are removed. Specifically, we observed significant importance of the "drug-target" and "target-target" edge types in DTI prediction, while other types of edges play a supporting role. This finding is correlated with the frequencies of different edge types in the adaptive meta-graph shown in Figure 4b. These results emphasize the importance of considering different edge types in meta-graph analysis to better understand the mechanisms of DTI prediction. We believe that these findings have significant implications for our research and further studies in the field.


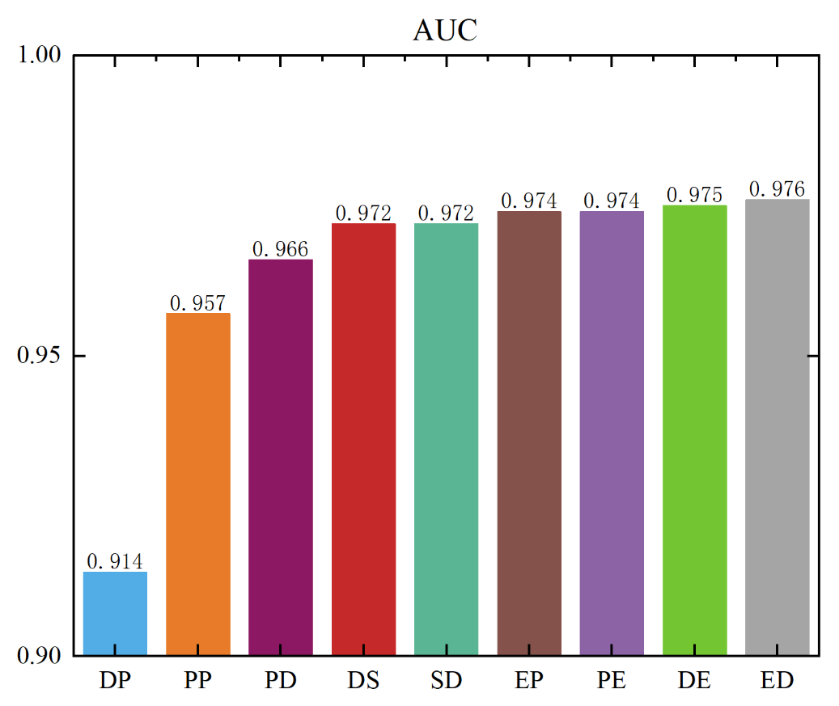


**Figure S2.** The AUC value is calculated to evaluate the performance of AMGDTI in predicting DTI after removing various edge types.

**S1.3 Cold start analysis**

We conducted a cold start analysis on two datasets as the reference^4^. Specifically, we firstly categorized 'cold' drug and protein pairs into three groups based on the numbers of their interactions, i.e., [0,3], (3,5] in Luo’s dataset and [0,3], (3,5], (5,10] in Zheng’s dataset. We investigated various cold-start degrees and designed corresponding cold-start nodes. In this experiment, 10 times of 5-fold cross-validation are performed on two benchmark datasets. In all these test scenarios, we maintain a balanced ratio of 1:1 between positive and negative samples. As we envisioned, it's evident that the first group presents the most challenging scenario, since drug and protein from this group have fewest contextual information. The experimental results are shown in Table S2.

According to the experimental results, the AMGDTI algorithm does not significantly decrease the prediction of DTI results in cold-start situations. Therefore, it proves that analyzing heterogeneous networks based on the adaptive meta-graph algorithm can fully utilize the diverse contextual information in the heterogeneous network, thus accurately predicting drug-target interactions with less information.

**Supplementary Table 2.** Results of cold start analysis in Luo’s and Zheng’s DTI data, where N represents the number of interactions.

|  | **Luo's dataset** | |  | **Zheng's dataset** | | |
| --- | --- | --- | --- | --- | --- | --- |
|  | **0<=N<=3** | **3<N<=5** |  | **0<=N<=3** | **3<N<=5** | **5<N<=10** |
| **AUC** | 0.919 | 0.947 |  | 0.923 | 0.942 | 0.94 |
| **AUPR** | 0.917 | 0.924 |  | 0.911 | 0.935 | 0.934 |

**S2. References**

1. Yunan Luo, Xinbin Zhao, Jingtian Zhou, Jinglin Yang, Yanqing Zhang, Wenhua Kuang, Jian Peng, Ligong Chen, and Jianyang Zeng. A network integration approach for drug-target interaction prediction and computational drug repositioning from heterogeneous information. Nature communications, 8(1):1–13, 2017.
2. Yi Zheng, Hui Peng, Xiaocai Zhang, Xiaoying Gao, and Jinyan Li. Predicting drug targets from heterogeneous spaces using anchor graph hashing and ensemble learning. In 2018 International Joint Conference on Neural Networks (IJCNN), pages 1–7. IEEE, 2018.
3. Adam Paszke, Sam Gross, Francisco Massa, Adam Lerer, James Bradbury, Gregory Chanan, Trevor Killeen, Zeming Lin, Natalia Gimelshein, Luca Antiga, et al. Pytorch: An imperative style, high-performance deep learning library. Advances in neural information processing systems, 32, 2019.
4. Y. Li, G. Qiao, X. Gao, and G. Wang, ‘Supervised graph co-contrastive learning for drug-target interaction prediction’, *Bioinformatics*, vol. 38, no. 10, pp. 2847–2854, May 2022, doi: 10.1093/bioinformatics/btac164.
